# Novel skin chamber for rat ischemic flap studies in regenerative wound repair

**DOI:** 10.1186/s13287-016-0333-0

**Published:** 2016-05-17

**Authors:** Yuan-Yu Hsueh, Duo-Hsiang Wang, Tzu-Chieh Huang, Ya-Ju Chang, Wei-Chi Shao, Tai-Lan Tuan, Michael W. Hughes, Chia-Ching Wu

**Affiliations:** Division of Plastic Surgery, National Cheng Kung University Hospital, College of Medicine, National Cheng Kung University, Tainan, Taiwan; Institute of Clinical Medicine, National Cheng Kung University Hospital, College of Medicine, National Cheng Kung University, Tainan, Taiwan; International Research Center for Wound Repair and Regeneration, College of Medicine, National Cheng Kung University, Tainan, Taiwan; Department of Cell Biology & Anatomy, National Cheng Kung University, Tainan, Taiwan; Institute of Basic Medical Sciences, National Cheng Kung University, Tainan, Taiwan; Department of Occupational Therapy, National Cheng Kung University, Tainan, Taiwan; The Saban Research Institute of Children’s Hospital Los Angeles and Department of Surgery, Keck school of Medicine, University of Southern California, Los Angelas, CA USA; Department of Biomedical Engineering, National Cheng Kung University, Tainan, Taiwan; Medical Device Innovation Center, National Cheng Kung University, No. 1, University Rd., Tainan, Taiwan

**Keywords:** Skin flap, McFarlane flap, Skin flap chamber, Diabetes, Cell therapy

## Abstract

**Background:**

In plastic surgery, skin flap is an important approach to reconstructive wound repairs. The rat dorsal skin flap is a clinically relevant and popular animal model to investigate and evaluate flap survival and necrosis. Nonetheless, flap survival is often unstable with unpredictable outcomes, regardless of previous attempts at design modification.

**Methods & Results:**

In the present study, we report a novel flap chamber that provides stable and reproducible outcomes by separating the dorsal skin flap from its surrounding skin by in situ immobilization. The flap chamber blocks circulation that disturbs flap ischemia from both basal and lateral sides of the flap tissue. Demarcation of skin necrosis is macroscopically evident on the flap and supported by distinct changes in histological architecture under microscopic examination. The utility of the novel skin flap chamber is further proven by applying it to the examination of flap survival in streptozotocin-induced diabetic rats with an increase in skin necrosis. The flap chamber also affords size modifications where a narrower flap chamber increases ischemia and provides manipulable therapeutic windows for studying cell therapies. Accordingly, intradermal injection of endothelial cells 3 days before flap ischemia significantly increases the survival of skin flaps.

**Conclusions:**

The novel flap chamber not only may stabilize the skin flap and provide reproducible outcomes that overcome the shortfalls of the traditional ischemic flap but also may afford size modifications that support research designs and test therapeutic approaches to regenerative repair.

**Electronic supplementary material:**

The online version of this article (doi:10.1186/s13287-016-0333-0) contains supplementary material, which is available to authorized users.

## Introduction

Surgical skin flaps are often used to repair wounds resulting from trauma, congenital defects, tumor excision, or other causes. Partial skin flap necrosis is a common problem in the clinic, especially at the distal part of the flap [1]. Flap necrosis is caused primarily by inadequate blood perfusion or ischemia-reperfusion and induces several detrimental changes in the tissue and vasculature, such as reactive oxygen species and superoxide dismutase activities [2]. Common ischemic factors such as poor surgical technique and handling, smoking, and diabetes mellitus may jeopardize skin flap survival. Management of the necrotizing flap, which is very time-consuming, requires repetitive dressing changes and in some cases a secondary reconstructive procedure. Therefore, the augmentation of perfusion in surgical flaps has long been a challenging clinical goal.

To explore novel mechanisms of blood vessel formation and interacting signals during tissue hypoxia, an animal model is needed to create a reproducible ischemic gradient in surgical flaps [3]. The dorsal rat flap model, first described by McFarlane et al. in 1965, is one of the most frequently used models to study angiogenesis and ischemia-reperfusion [4]. Many approaches to enhance the survival of surgical flaps have been tested in the prevention of ischemic injuries, such as preconditioning with transient remote ischemia [5], vascular endothelial growth factor treatment [6], or platelet-rich plasma treatment [7]. The concepts of tissue engineering and regeneration were also proposed by applying various stem cell therapies to promote vasculogenesis in the ischemic flap [8–11]. However, the variability of flap necrosis leads to difficulties in comparative results between different therapeutic interventions [12]. The uncertain vascular distribution in the flap is one of the causes of necrosis variability. Modification of flap size according to rat size and the underlying bony landmarks is suggested to reduce this variation [13, 14]. Collateral vessel compensation from the surrounding skin and wound bed is an additional reason for the extensive variability [15].

Despite several modifications and standardization proposals to help to stabilize ischemic flap survival, the extent of flap necrosis is still highly variable (25 % to 50 % necrosis of flap length) [12, 14, 16]. A reliable flap model is therefore highly demanded, especially for cell therapies of the ischemic flap of either healthy or circulation-compromised skin. So far, there is no proposed flap modification that is designed to stabilize the dorsal skin flap and to isolate the ischemic skin flap from circulatory interference from the adjacent skin. To minimize the unpredictability of dorsal skin flap necrosis, we introduce an innovative flap chamber and verify its reproducibility in both healthy and disease models, including therapeutic approaches.

## Methods

### Animal housing and surgery for skin flap

All animal procedures complied with clause 16 of the Animal Protection Act, and all experiments were monitored and approved by Institutional Animal Care and Use Committee of National Cheng Kung University. All male Sprague-Dawley (SD) rats (8 weeks old, 270–300 g) were housed in groups of two in an environmentally controlled room, at consistent temperature and humidity, on a 12:12 h light/dark cycle, with food and water ad libitum.

All surgeries were conducted by the same surgeon and followed an identical protocol. The SD rats were randomly divided into suture (n = 8) or chamber model (n = 8) groups, depending on the experimental treatment. Surgical anesthesia was induced and maintained by intraperitoneal injection of Zoletil® (Virbac, Carros, France) at a dosage of 50 mg/kg. After anesthesia, the surgical area of dorsal skin was shaved and sterilized with 70 % ethanol. To compare the common skin flap model with the new developed chamber, the McFarlane flap was created in the suture group according to previous studies [4, 16]. Briefly, the three-sided full-thickness skin flap (3.6 × 7.2 cm in original and 3 × 10 cm in narrow ones) was created on the dorsal skin of rats by raising the skin from the iliac crest to the scapular tip by using sharp dissection with scissors. The three-sided island skin flap included skin, subcutaneous tissue, and panniculus carnosus muscle. A silicon membrane adjusted to the flap size was placed beneath the flap as a barrier to prevent neovascularization from the wound bed perturbing the flap. The flap was sutured in situ to the surrounding wound edge by 4-0 polypropylene sutures. The body weight of rats slightly increased with normal activities after flap chamber implantation.

### Development of new skin flap chamber

**Fig. 1:**
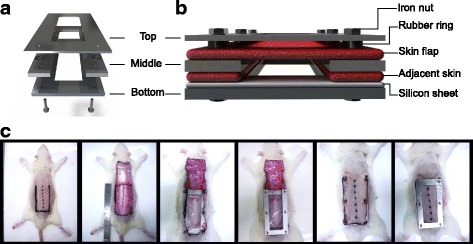
Flap chamber composition and step-by-step assembly. **a** Three components of chamber. **b** The relationship between dorsal skin flap and the flap chamber. **c** The protocols for step-by-step flap chamber assembly: marking of dorsal skin flap, raising of the full-thickness skin flap, implantation of the bottom plate, placement of the middle plate, fixation of skin flap above the middle plate with rubber ring protection, covered by top plate with iron nut fixation

To ameliorate the unpredictable flap survival pattern and ratio in the traditional McFarlane flap, we designed an innovative flap chamber to immobilize the ischemic flap to the adjacent dorsal skin. The chamber was assembled by top, middle, and bottom layers by using a stainless plate with screws and nuts (Fig. 1a, detailed scale in Additional file 1: Figure S1).

The process used in the suture group was repeated in the chamber group to create three-sided skin flaps. The adjacent skin was fixed between the middle and bottom plates by inserting a silicon membrane beneath the flap (Fig. 1b). The elevated three-sided ischemic flap was then secured between the top and middle plates to separate the ischemic flap from the adjacent skin. The detail of the ischemic flap chamber assembly with skin flap model setup is described as follows (Fig. 1c): after flap elevation, a bottom plate with a silicon membrane adjusted to size was implanted, the adjacent skin flap was punched by a 2-mm punch biopsy and screws inserted to stabilize the base of the chamber, a middle plate was placed on the adjacent skin edge, the skin flap was then punched and secured above the middle plate, rubber gasket rings were placed above the skin flap to avoid pressure loading on the skin flap from the top plate of chamber, and finally the top plate was positioned onto the skin flap and fixed by steel nuts. After the surgical procedures, all rats were positioned under a warming lamp until conscious. Animals were sacrificed by overdose of carbon-dioxide inhalation and the skin tissue harvested after post-operation day 7 (POD7).

### Diabetic state induction

After 48 h of fasting, SD rats (270 to 300 g) were intraperitoneally injected with a single shot of streptozotocin (STZ) (Sigma-Aldrich, St. Louis, MO, USA) at a dosage of 70 mg/kg. The STZ was freshly prepared by dissolving in 50 mM sodium citrate buffer (pH = 4.5) prior to injection. The diabetic state was confirmed by monitoring the elevation of blood glucose to more than 250 mg/dl with fasting for 8 h after the STZ induction for 21 days.

### Cell therapy in the skin flap chamber

Human umbilical vein endothelial cell (HUVEC) administration was used as a salvage therapy in ischemic tissue [11]. The protocol for the isolation of HUVECs has been described previously [17]. In brief, human umbilical cords were washed with normal saline to remove blood clots. The umbilicus vein was perfused with 0.75 % type I collagenase (Invitrogen, Waltham, MA, USA) to isolate HUVECs from vessel walls and cultured in M199 medium (Invitrogen) supplemented with 20 % fetal bovine serum (HyClone, part of GE Healthcare, Little Chalfont, UK). To study the enrichment of endothelial cells in preventing flap necrosis, the HUVECs were transplanted by intradermal injection 3 days prior to flap surgery [9, 10]. A total number of 1 × 10^6^ cells was suspended in 1 ml of normal saline and evenly injected into the intradermal layers of skin along the middle axis.

### Necrosis and histological assessment

To observe the gross changes due to skin necrosis, photographs of skin flaps were taken by using a digital camera (IXUS-105, Canon, Tokyo, Japan) at the same focal distance under 3 % isoflurane anesthesia (Panion & BF Biotech Inc., Taipei, Taiwan) on PODs 3, 5, and 7. After the rats were sacrificed, the necrotic edge of the skin flap was judged by the skin’s appearance, consistency, and the absence of blood flow perfusion [18]. An area of the flap was assigned as a necrotic part if it exhibited a darker surface, abnormal stiffness, and lack of blood flow after being cut by scissors. After the necrotic edge was judged, the viable area of the flap was selected and quantified by using ImageJ software (version 1.47 t, Wayne Rasband, National Institutes of Health, Bethesda, MD, USA). The ratio of viable to non-viable skin flap was calculated as the viable area as a percentage of the necrotic area in the entire skin flap.

The harvested skin tissues were fixed with 4 % paraformaldehyde (Sigma-Aldrich), then dehydrated in a gradient series of ethanol solutions and finally xylene cleared and embedded in paraffin. The samples were sectioned longitudinally to a thickness of 10 μm and stained with hematoxylin and eosin (H&E) to observe tissue morphology. The immunohistochemical (IHC) stainings were used to determine vascular structures and protein expressions by using specific antibodies in accordance with the established protocols [19]. The primary antibodies with specific dilution ratio were used for von Willebrand factor (vWF) (1:100, Santa Cruz Biotechnology, Inc., Dallas, TX, USA), alpha-smooth muscle actin (α-SMA) (1:500, Abcam, Cambridge, UK), and B-cell lymphoma 2 (Bcl2, 1:100, Santa Cruz Biotechnology, Inc.). The digital images were taken with the same microscope (BX51, Olympus, Tokyo, Japan).

### Statistics

The data are expressed as the mean ± standard deviation. Parametric analysis was performed for comparisons between the experimental groups. The Student’s *t* test was used for the survival ratio of flaps and for fasting blood glucose. Multiple comparisons among groups were performed by using one-way analysis of variance followed by the Bonferroni post hoc test. *P* values of less than 0.05 were considered to indicate statistical significance. All statistical analyses were performed by using graphing and analysis software (Origin version 8.5, OriginLab Corporation, Northampton, MA, USA).

## Results

### Variable and unstable survival using the traditional McFarlane flap

**Fig. 2:**
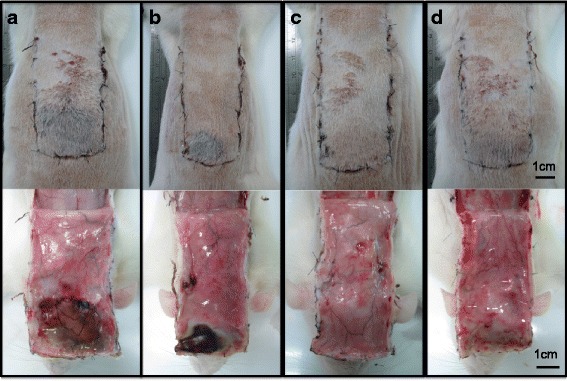
Common discrete patterns of flap survival in suture model (flap size of 3.6 × 7.2 cm). Round shape skin necrosis (**a**). Unilateral skin necrosis (**b**). Absent skin necrosis (**c**). Unclear epidermal demarcation line (**d**)

The survival ratio from the McFarlane flap revealed highly variable survival with an unstable pattern 7 days after surgery (Fig. 2). The necrotic regions of the distal flaps either were located in the center (Fig. 2a) or were crooked (Fig. 2b). Moreover, the survival area ranged from 80 % to 100 % (Fig. 2a-d). After flap elevation, some rats showed a clear demarcated line between the survival and necrotic areas (Fig. 2a,b) whereas other rats did not distinguish a clear necrotic area (Fig. 2d). Lack of a clear demarcation increases the difficulty in calculating the survival ratio. In addition, the contraction of the flap was present in most cases of the McFarlane flap and exhibited variable distorted patterns with different degrees of flap necrosis.

### Highly stabilized ischemic model by flap chamber

**Fig. 3:**
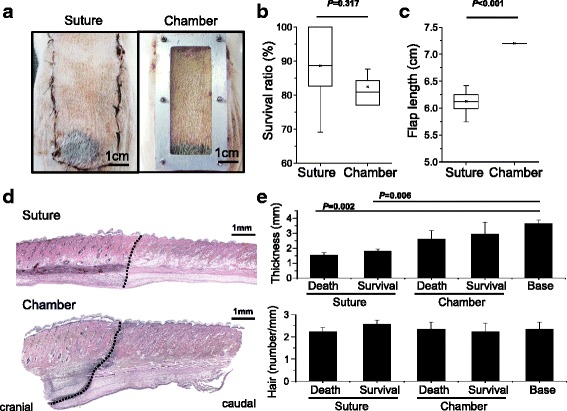
Reproducible skin survival by flap chamber. Gross picture of flap survival between suture and chamber model (**a**). The box plot of survival ratio distribution of dorsal flap (n = 8 in each group) (**b**). The skin contraction decreased the flap length in the suture model but not in the flap chamber (**c**). Hematoxylin-and-eosin staining from cranial to caudal, across the skin necrosis line (*black dotted line*) (**d**). Quantification of skin thickness and number of hair follicles were compared from cranial to caudal survival and death region (**e**)

The flap chamber achieved more stable results compared with the traditional suture model in the McFarlane flap (Fig. 3a). From the distal to the proximal flap, the necrotic skin showed a linear gradient of ischemic pattern. The quantification of the survival area in the flap chamber demonstrated highly repeatable skin necrosis with a small variation when assembling the three-sided full-thickness skin in the flap chamber (Fig. 3b). The quantification of flap length revealed a high degree of skin contraction that distorted and shortened the survival area in the flap from the suture model, and this could be prevented by using the flap chamber device; that is, there was no flap length change in the chamber group (Fig. 3c). In the longitudinal section of skin histology using H&E staining (Fig. 3d), the flap chamber provided a clear demarcation line and distinct microscopic architecture between the proximal survival (cranial) skin and the distal death (caudal) region. The full thicknesses of both survival and death skin were significantly decreased in the suture model, as compared with the cranial base area. There were no significant changes of skin thickness in both the survival and death regions in the chamber model (Fig. 3e). The morphology of hair follicles became apoptotic and shrunk in the death region of the ischemic flap without a significant difference in the number of hair follicles. The image data also demonstrated normal architecture of full-thickness skin in the survival region of the flap chamber when compared with the suture model.

### Distinct pattern of vasculature across the demarcation line by flap chamber

**Fig. 4:**
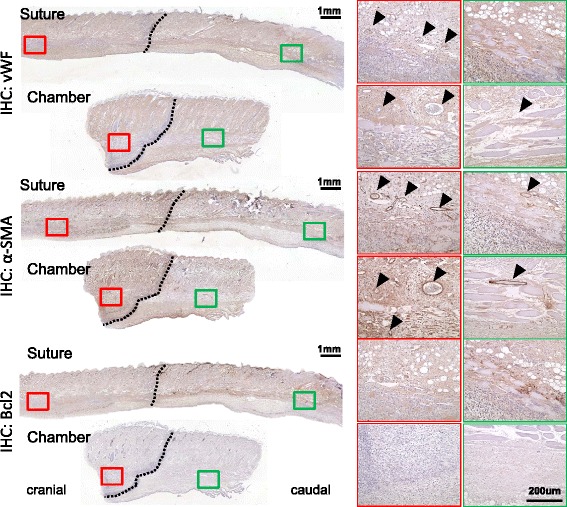
Immunohistochemical (*IHC*) stain of vascular pattern across the demarcation line. The IHC stain of both von Willebrand factor (*vWF*) and alpha-smooth muscle actin (*α-SMA*) revealed increased signals in the cranial region as compared with the caudal region (*black arrowhead*: positive staining). No positive staining of B-cell lymphoma 2 (*Bcl2*) was observed across the demarcation line

To verify the pattern of vasculature across the demarcation line, the IHC stain of vWF and α-SMA revealed a distinct difference of lumen number and vascular structures across the demarcation line in the chamber model (Fig. 4). A similar difference in vasculature could be found in suture model, but with a longer distance across the demarcation line. There was no difference in Bcl2 signal across the demarcation line.

### Application of flap chamber in a disease model

**Fig. 5:**
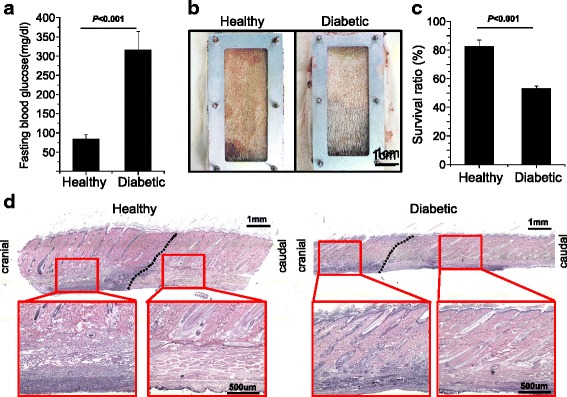
Flap survival in streptozotocin (*STZ*)-induced diabetic rats. Significant increase of blood sugar indicates a successful diabetic model in fasting rats 3 weeks after STZ induction (**a**). Gross picture of flap survival between healthy and diabetic rats (n = 4 in each group) (**b**). Diabetes significantly decreased the flap survival ratio after flap surgery for 7 days (**c**). The histological staining of skin architecture in healthy and diabetic flaps (**d**). Data are expressed as mean ± standard deviation

The stable results from the flap chamber indicate the possibility of further investigating the necrosis of skin flap in a diseased animal. Diabetic status was successfully induced by intraperitoneal injection of STZ into adult rats and showed an elevation of blood sugar 3 weeks after induction (Fig. 5a). When the flap chamber in diabetic rats was used, increases in the necrosis area were observed 7 days after surgery (Fig. 5b). The survival ratio of the skin flap was significantly decreased by approximately 50 % compared with healthy, non-diabetic rats (Fig. 5c). As compared with the healthy group (Figs. 3d and 5d), the longitudinal section of skin histology using H&E staining also revealed distinct differences in skin architecture, skin thickness, and morphology of hair follicles between the survival and necrotic regions of the flap from diabetic rats (Fig. 5d). Consistent flap survival in the diabetic rat model could permit a further survey of flap physiology or the application of potential clinical therapeutic modalities.

### Chamber modification for cell therapy

**Fig. 6:**
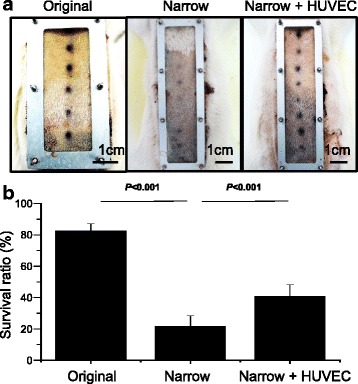
Modification of flap chamber for cell therapy. Gross pictures for flap survival between original (3.6 × 7.2 cm, 20 g), narrow (3 × 10 cm, 17 g), and cell therapy in narrow chamber by human umbilical vein endothelial cells (*HUVECs*) (Narrow + HUVEC) (**a**). The increase of flap necrosis in the narrow chamber was salvaged by application of HUVECs (n = 4 in each group) (**b**). Data are expressed as mean ± standard deviation

The initial design of the flap chamber was developed to scale (3.6 × 7.2 cm for a 1:2 width-to-length ratio, 20 g in chamber weight) to achieve an average of 20 % necrosis and to compare its stability with the McFarlane suture using the same flap size. After confirmation that stable survival using the flap chamber could be achieved, further versatile design modifications were made to increase the challenge of therapeutic window for advanced purposes in cell therapies. By changing the chamber size to a narrow scale (3 × 10 cm for over 1:3 in width-to-length ratio, 17 g in chamber weight), an average necrosis area of 80 % was created in the age-matched rats as compared with the initial flap chamber design (Fig. 6a). The skin flap demarcation line remained clear in the narrow flap chamber. HUVECs from human umbilical cord blood consisted of late endothelial progenitor cells, which contributed to angiogenesis and protected the tissue from ischemia in our previous study [17]. HUVECs were equally implanted into the dermis of the skin flap for 3 days before flap surgery (Fig. 6a, *black spots* in Narrow + HUVEC rat). With the treatment of cell therapy, the extent of flap survival could be significantly salvaged to 40 %, as compared with 20 % skin survival in the narrow flap chamber group (Fig. 6b). The versatile property and tight control of the percentage of skin survival using these flap chambers enabled researchers to study the different degrees of injury. These results provide solid evidence that the newly developed flap chamber is an ideal platform to provide a consistent in vivo microenvironment for skin necrosis, which is important for investigating the underlying ischemic molecular signals and decreasing the numbers of animals and therapeutic cells used in future studies.

## Discussion

After the initial description of the rat ischemic flap model by McFarlane et al*.* in 1965, there were numerous studies using the “McFarlane flap” to study the “delay phenomenon” or critical flap survival [10, 20–22]. Several modifications were adopted to decrease the uncertainty of flap survival, such as varying flap dimensions from 2 × 9 cm to 3 × 10 cm, raising the flap from either the cranial or caudal end, or changing the flap according to different anatomical (bony) landmarks. All of these attempts were to minimize the large variation in flap survival (from 50 % to 75 % in the traditional McFarlane flap). The most significant feature of these modifications focused on the relationship of the flap to the underlying anatomical structure by inclusion or exclusion of direct cutaneous or musculocutaneous vessels in the flap [12]. In the McFarlane or modified McFarlane flap, one silicon membrane was placed underneath the skin flap to prevent neovascularization from the underlying wound bed. However, the neovascularization from the skin suture edge might also be established from collateral vessel formation, resulting in disturbance of the necrotic pattern and an unpredictable flap survival [3].

When the flap chamber was used, the elevated three-sided flap was fixed and separated from the underlying wound bed and the adjacent skin (Fig. 1). This manner ensured that the skin flap was supplied by one side of the flap pedicle cranially. The survival pattern at the distal portion of the flap was more stable in a linear fashion, from cranial to caudal (Fig. 3a,b). A clear demarcation line could be observed not only macroscopically but also microscopically in histology, which could not be observed in the “traditional” suture method. In addition, the architecture of skin and skin appendage was apparently different across the demarcation line histologically (Fig. 3d,e). In addition, the clear demarcation line of skin necrosis in the flap chamber model revealed a distinct decrease of vascular density immediately across the demarcation line by IHC stain of vWF and α-SMA (Fig. 4). The suture model did not show such dramatic change of vasculature across the demarcation line.

This chamber could be used to stabilize the outcome in disease models, such as a diabetic flap model. It is well known that diabetes impairs ischemia-driven neovascularization and causes fewer collateral circulation vessels in both animal and human tissues [23, 24]. Any confounder of such fragile microcirculation in the diabetic ischemic flap might lead to widely variable results. When the flap chamber in a diabetic ischemic skin flap was used, consistent flap survival of 50 % could be achieved, a significant difference from the control model (Fig. 5b,c). Our chamber provided similar histology results in the diabetic skin flap and revealed homogenous atrophy in both the hypodermal and dermal layers (Fig. 5d), including significant reductions in capillary density and dermal thickness that agreed with skin necrosis reported in previous studies [25, 26]. In addition, the demarcation line was seen very clearly, with no difficulty in evaluating the survival grossly, as compared with the vague, uneven distribution in the traditional McFarlane flap in diabetic rats [25, 26].

To apply the ischemic flap model to various types of projects, a versatile modification could easily be made while maintaining stability. As for the field of stem cell therapy research, the ideal therapeutic window could be created simply by changing the scale of the chamber design. As shown in Fig. 6, changing the chamber from the original dimensions (3.6 × 7.2 cm) to a narrower (3 × 10 cm) feature could significantly decrease the flap survival from 80 % to 20 %. By implantation of HUVECs 3 days before surgery, the ischemic flap could be salvaged significantly from 20 % to 40 %. This result demonstrated that by using the flap chamber, the flap survival was predictable with or without stem cell therapies. The dimension of the flap chamber was versatile and could be modified to create different therapeutic windows for corresponding stem cells to treat.

## Conclusions

This novel ischemic flap chamber could stabilize the results of variable skin necrosis in animal studies of the traditional McFarlane flap. By changing the scale in width-to-length ratio of this chamber, it is made to fit well to both the healthy and disease models of ischemic flaps. In addition, it is feasible to apply to the model to stem cell therapy with stable therapeutic outcomes.

## Additional file

Additional file 1: Figure S1. Detailed scale for three-layer flap chamber in different width-to-length ratio. (PPTX 535 kb)

## Abbreviations

H&E: hematoxylin and eosin
HUVEC: human umbilical vein endothelial cell
IHC: immunohistochemic
POD: post-operation day
SD: Sprague-Dawley
STZ: streptozotocin
vWF: Von Willebrand factor
α-SMA: alpha smooth muscle actin
