# Supplementary figures and images for: Novel skin chamber for rat ischemic flap studies in regenerative wound repair
Source: Stem Cell Res Ther. 2016 May 17;7:72. doi: 10.1186/s13287-016-0333-0 (PMC4869367; doi:10.1186/s13287-016-0333-0)

## Slide 1
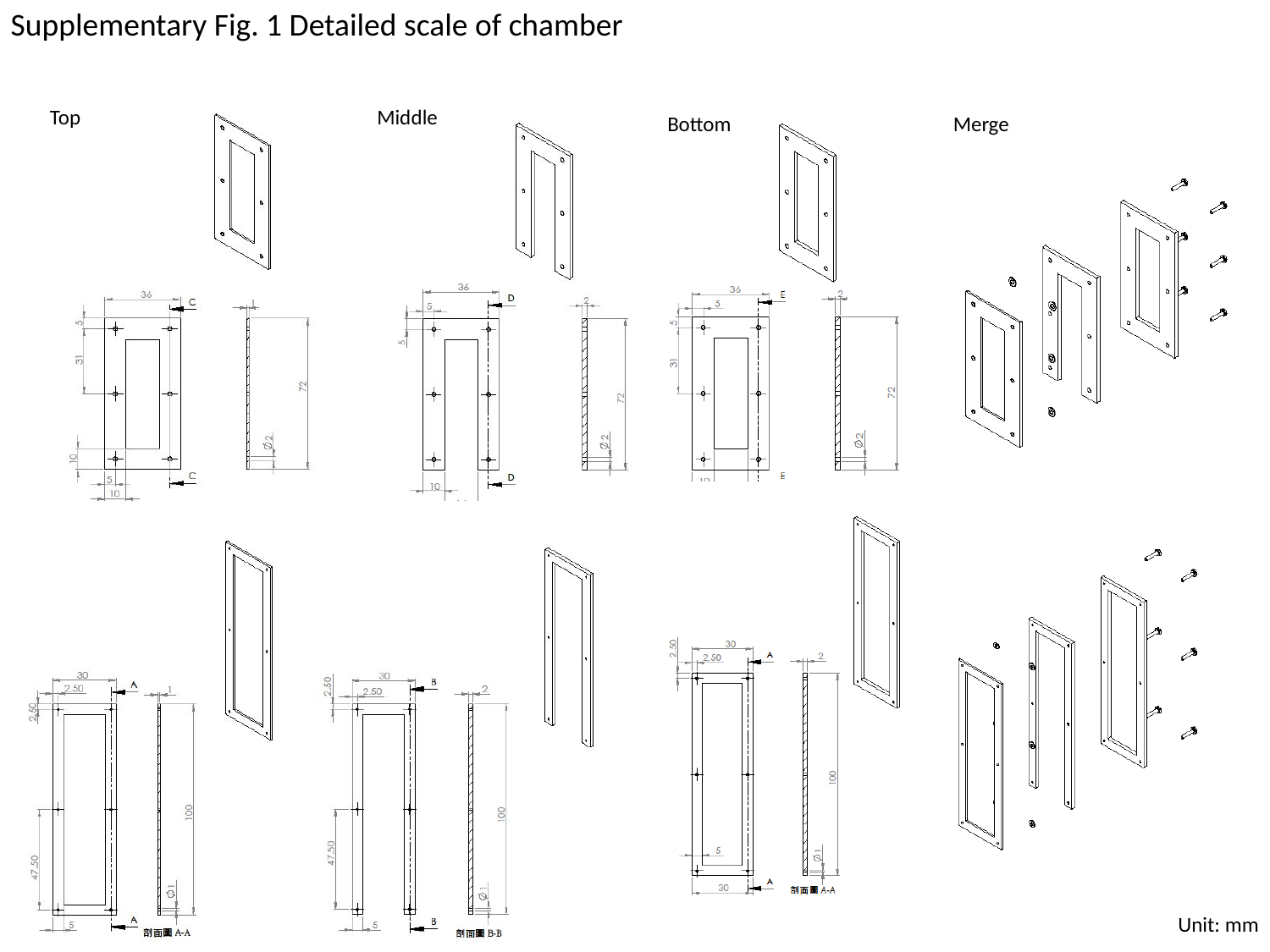

Supplementary Fig. 1 Detailed scale of chamber
Top
Middle
Bottom
Merge
3.6*7.2
3*10
Unit: mm

Supplement: Additional file 1: Figure S1. — Detailed scale for three-layer flap chamber in different width-to-length ratio. (PPTX 535 kb) [file 13287_2016_333_MOESM1_ESM.pptx]
